# Nitrate enhances skeletal muscle fatty acid oxidation via a nitric oxide-cGMP-PPAR-mediated mechanism

**DOI:** 10.1186/s12915-015-0221-6

**Published:** 2015-12-22

**Authors:** Tom Ashmore, Lee D. Roberts, Andrea J. Morash, Aleksandra O. Kotwica, John Finnerty, James A. West, Steven A. Murfitt, Bernadette O. Fernandez, Cristina Branco, Andrew S. Cowburn, Kieran Clarke, Randall S. Johnson, Martin Feelisch, Julian L. Griffin, Andrew J. Murray

**Affiliations:** Department of Physiology, Development & Neuroscience, University of Cambridge, Downing Street, Cambridge, CB2 3EG UK; Department of Biochemistry, University of Cambridge, Cambridge, UK; MRC-Human Nutrition Research, University of Cambridge, Cambridge, UK; Faculty of Medicine, Clinical & Experimental Sciences, University of Southampton, Southampton, UK; Department of Physiology, Anatomy & Genetics, University of Oxford, Oxford, UK

**Keywords:** Fatty acid oxidation, Metabolism, Mitochondria, Muscle, Nitrate, Nitric oxide

## Abstract

**Background:**

Insulin sensitivity in skeletal muscle is associated with metabolic flexibility, including a high capacity to increase fatty acid (FA) oxidation in response to increased lipid supply. Lipid overload, however, can result in incomplete FA oxidation and accumulation of potentially harmful intermediates where mitochondrial tricarboxylic acid cycle capacity cannot keep pace with rates of β-oxidation. Enhancement of muscle FA oxidation in combination with mitochondrial biogenesis is therefore emerging as a strategy to treat metabolic disease. Dietary inorganic nitrate was recently shown to reverse aspects of the metabolic syndrome in rodents by as yet incompletely defined mechanisms.

**Results:**

Herein, we report that nitrate enhances skeletal muscle FA oxidation in rodents in a dose-dependent manner. We show that nitrate induces FA oxidation through a soluble guanylate cyclase (sGC)/cGMP-mediated PPARβ/δ- and PPARα-dependent mechanism. Enhanced PPARβ/δ and PPARα expression and DNA binding induces expression of FA oxidation enzymes, increasing muscle carnitine and lowering tissue malonyl-CoA concentrations, thereby supporting intra-mitochondrial pathways of FA oxidation and enhancing mitochondrial respiration. At higher doses, nitrate induces mitochondrial biogenesis, further increasing FA oxidation and lowering long-chain FA concentrations. Meanwhile, nitrate did not affect mitochondrial FA oxidation in PPARα^−/−^ mice. In C2C12 myotubes, nitrate increased expression of the PPARα targets *Cpt1b*, *Acadl*, *Hadh* and *Ucp3*, and enhanced oxidative phosphorylation rates with palmitoyl-carnitine; however, these changes in gene expression and respiration were prevented by inhibition of either sGC or protein kinase G. Elevation of cGMP, via the inhibition of phosphodiesterase 5 by sildenafil, also increased expression of *Cpt1b*, *Acadl* and *Ucp3*, as well as CPT1B protein levels, and further enhanced the effect of nitrate supplementation.

**Conclusions:**

Nitrate may therefore be effective in the treatment of metabolic disease by inducing FA oxidation in muscle.

**Electronic supplementary material:**

The online version of this article (doi:10.1186/s12915-015-0221-6) contains supplementary material, which is available to authorized users.

## Background

As the largest insulin-sensitive tissue in the body, skeletal muscle plays a vital role in maintaining glucose homeostasis via the uptake, storage and oxidation of carbohydrate during the postprandial period. Under fasting conditions, however, fatty acid (FA) oxidation dominates to satisfy muscle energy needs whilst sparing glucose and protecting muscle glycogen reserves [1]. Such flexibility in fuel selection is a hallmark of metabolically-healthy muscle; indeed, in human muscle, insulin sensitivity relates strongly to a capacity to increase FA oxidation in the presence of fat [2]. Furthermore, the expression of genes involved in oxidative phosphorylation [3] and FA oxidation [4] are downregulated in diabetic muscle. The atypical accumulation of intramyocellular lipids, possibly resulting from impaired FA oxidation, has been associated with the aetiology of insulin resistance [5].

Promotion of muscle FA oxidation thus emerged as a strategy for the treatment of insulin resistance [6], with the peroxisome proliferator-activated receptor (PPAR) transcription factors representing key targets [7]. PPARα is expressed in liver, heart and skeletal muscle, and when activated increases the expression of FA oxidation genes [8]. Meanwhile, PPARβ/δ is ubiquitously expressed, abundantly present in skeletal muscle [8] and, when stimulated, can reduce obesity and improve insulin sensitivity [9, 10]. In high-fat feeding models, however, enhanced PPAR target gene expression was associated with increased rates of incomplete FA oxidation and intra-mitochondrial accumulation of potentially harmful intermediates, e.g. acyl-CoAs and acyl-carnitines [11]. Indeed, in mice lacking malonyl-CoA decarboxylase (MCD), decreased mitochondrial FA import and catabolism were associated with protection against glucose intolerance [11].

The concept of mitochondrial overload was thus proposed, in which increased lipid supply and β-oxidation is not accompanied by sufficient tricarboxylic acid cycle activity to support complete FA oxidation [12]. PPARγ co-activator 1α (PGC-1α), a transcriptional co-regulator that interacts with the PPARs, is considered the master regulator of mitochondrial biogenesis [13]. Increased PGC-1α expression following exercise-training prevented lipid-overload-induced mitochondrial deficiency associated with high-fat feeding, lowering muscle concentrations of long-chain acyl-carnitines [14].

Recently, a role has emerged for cyclic guanosine monophosphate (cGMP) in regulating energy metabolism [15]. In muscle, cGMP stimulated mitochondrial biogenesis and FA oxidation [16], whilst in white adipose tissue (WAT), cGMP, acting via cGMP-dependent protein kinases (PKGs) promoted the browning response characterised by increased mitochondrial density and uncoupling protein 1 (UCP1) expression [17, 18].

Dietary nitrate increases plasma cGMP in humans [19], whilst nitrite increased cGMP levels in some tissues [20]. Nitrate reversed features of metabolic syndrome in eNOS^−/−^ mice, including a reduction in body fat and improved glucose homeostasis and insulin sensitivity [21]. Recently, we showed that inorganic nitrate promoted the browning of WAT via activation of soluble guanylate cyclase (sGC) [22]. Moreover, we found that nitrate prevented the downregulation of FA oxidation in the hypoxic rat heart [23]. The hypoxic suppression of FA oxidation is commonly seen in both human and rodent skeletal muscle [24] as well as rat heart [25], and may result from the hypoxia-inducible factor (HIF)-dependent suppression of PPARα transcriptional activity and/or expression, as seen in some tissues [26]. FA oxidation requires more O_2_ than glucose oxidation (per ATP synthesised) and thus this mechanism may spare O_2_ in hypoxic tissues. The preservation of FA oxidation in the hypoxic heart following nitrate supplementation may be an indirect effect arising from improvements in blood flow and therefore tissue oxygenation, but notably nitrate also increased the FA oxidation capacity of cardiac mitochondria in normoxic rats [23], perhaps suggesting a direct effect on metabolic regulation.

We therefore investigated the possible effects of dietary nitrate on FA oxidation in skeletal muscle, hypothesising that nitrate would enhance mitochondrial biogenesis and β-oxidation. We found that nitrate enhances skeletal muscle FA oxidation in rodents in a dose-dependent manner. Nitrate induces FA oxidation through a sGC/cGMP-mediated PPARβ/δ- and PPARα-dependent mechanism. Enhanced PPARβ/δ and PPARα expression and DNA-binding induces expression of FA oxidation enzymes, increasing muscle carnitine and lowering tissue malonyl-CoA concentrations, thereby supporting intra-mitochondrial pathways of FA oxidation and enhancing mitochondrial respiration. At higher doses, nitrate also induces mitochondrial biogenesis, further increasing FA oxidation and lowering long-chain FA concentrations.

## Results

### Dietary nitrate counteracts the hypoxic suppression of PGC-1α in skeletal muscle and enhances FA oxidation in both normoxic and hypoxic skeletal muscle

Since nitrate supplementation prevented the hypoxic suppression of FA oxidation in rat cardiac muscle [23], we investigated whether nitrate was similarly protective in the skeletal muscle of rats exposed to hypoxic conditions (13 % O_2_) for 14 days compared with rats housed in a normoxic environment. Rats were supplemented with either 0.7 mM sodium nitrate or 0.7 mM sodium chloride (controls) via their drinking water and maintained on a standardized quality-controlled (SQC) diet to normalize micronutrient intake. Nitrate concentrations of food and water were known, thus facilitating calculation of nitrate intake. No differences were observed in food intake or volumes of water consumed between any of the groups (data not shown). As shown previously, consumption of nitrate-supplemented water, increased nitrate intake by 5- to 7-fold compared with sodium chloride controls [23].

**Fig. 1:**
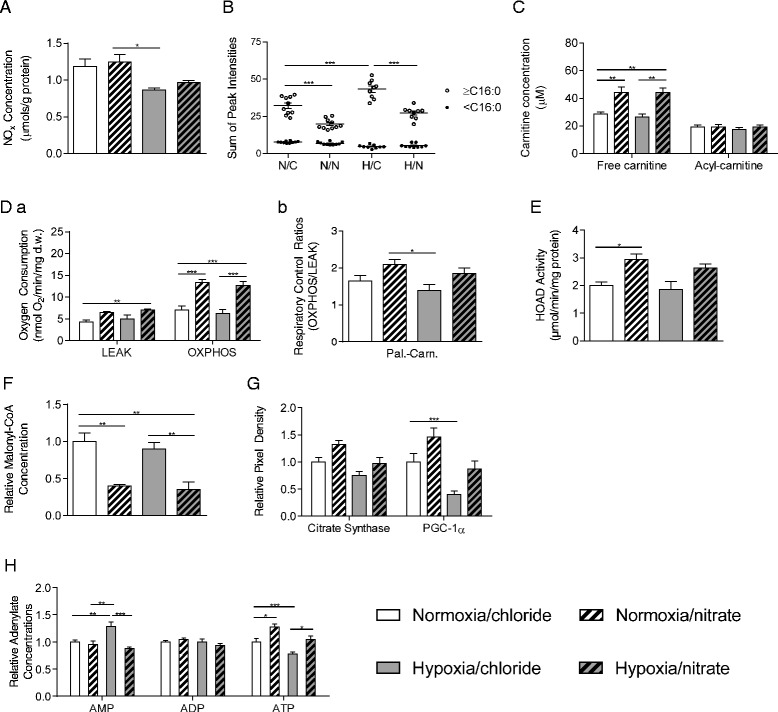
Dietary nitrate counteracts the hypoxic suppression of PGC-1α in skeletal muscle and enhances fatty acid oxidation in both normoxic and hypoxic skeletal muscle. (**a**) Soleus total nitrogen oxide levels (NOx); (**b**) Fatty acid profile of soleus tissue divided into ≥ C16 (longer-chain) and < C16 (shorter-chain); (**c**) Carnitine and acyl-carnitine profile of soleus; (**d**) Respiration rates and respiratory control ratios with palmitoyl-carnitine and malate substrates in permeabilized soleus muscle fibres; (**e**) 3-hydroxyacyl-CoA dehydrogenase activity in soleus tissue; (**f**) Malonyl-CoA levels in soleus tissue extracts; (**g**) Citrate synthase and PGC-1α protein levels in soleus tissue; (**h**) AMP, ADP and ATP levels in soleus tissue extracts. Data are represented as mean ± SEM, n = 4–10 per experimental group. * *P* ≤0.05; ** *P* ≤0.01; *** *P* ≤0.001

Nitrate supplementation increased plasma nitrate concentrations in both normoxic and hypoxic rats, as shown previously [23], with no change in nitrite concentrations. In soleus muscle, hypoxia decreased total NO_x_, and this was unaffected by dietary nitrate supplementation (Fig. 1a).

To understand whether nitrate supplementation altered FA oxidation in vivo, we measured total FA concentrations (free and conjugated) in whole skeletal muscle, and found that shorter-chain FAs (here defined as those < C16, i.e. the shortest FA provided by SQC diet) were the same in all groups, whilst longer-chain FAs (≥C16) decreased by 38 % upon nitrate supplementation, but increased by 34 % with hypoxic exposure (Fig. 1b). In nitrate-supplemented hypoxic rats, longer-chain FAs decreased by 37 % compared with non-supplemented hypoxic rats (Fig. 1b), and were comparable with normoxic rats receiving sodium chloride in their water.

Next, we measured carnitine and acyl-carnitine concentrations in the muscle of these rats. Total acyl-carnitine levels were the same in all groups. However, the concentration of free carnitine was increased by 54 % following nitrate supplementation, whether in normoxic or hypoxic rats, whilst hypoxia alone did not alter carnitine concentrations (Fig. 1c). In humans, carnitine loading supports enhanced muscle FA oxidation, preventing body fat accumulation [27]. An increased ratio of long chain acyl-carnitines to free carnitine has been described in the hypoxic rat heart in association with decreased β-oxidation [28], whilst Oram et al. [29] speculated that high intracellular concentrations of free carnitine (as found here in nitrate - treated rats) may be important for selectively channelling fat for oxidation rather than lipid biosynthesis.

To understand how hypoxia/nitrate affected the capacity of skeletal muscle mitochondria for FA oxidation, respiration rates were measured in permeabilized soleus muscle fibre bundles with a fatty acyl-carnitine substrate (0.04 mM palmitoyl-carnitine + 5 mM malate). LEAK state respiration (i.e. O_2_ consumption which is not coupled to ADP phosphorylation) appeared to be unaffected by either hypoxia or nitrate alone. However, nitrate supplementation in hypoxia resulted in LEAK state FA respiration rates that were 64 % higher than in normoxic/chloride rats (Fig. 1d). In contrast to our previous finding in heart [23], soleus OXPHOS state FA respiration rates were unaffected by hypoxia alone; however, in both normoxic and hypoxic rats, nitrate supplementation resulted in OXPHOS state FA respiration rates that were 90 % and 81 % higher, respectively, than in chloride-supplemented controls (Fig. 1d).

We therefore investigated two aspects of mitochondrial FA metabolism, namely activity of the β-oxidation enzyme, 3-hydroxyacyl-CoA dehydrogenase (HOAD), and tissue concentrations of malonyl-CoA, a negative regulator of mitochondrial FA import via its inhibitory effects on the carnitine palmitoyl-transferase (CPT) system. Whilst hypoxia did not affect either of these markers, nitrate supplementation increased HOAD activity in normoxic rats (Fig. 1e) and lowered tissue malonyl-CoA concentrations in both normoxic and hypoxic rats (Fig. 1f).

In addition to this, we aimed to understand whether the enhanced FA oxidation capacity following nitrate supplementation could be explained by changes in mitochondrial density. Protein levels of citrate synthase, a tricarboxylic acid cycle enzyme and marker of mitochondrial density, were not significantly altered by either hypoxia or nitrate, although there was a trend towards decreased levels in hypoxia that was prevented by nitrate, and a trend towards increased levels in normoxic rats with nitrate supplementation. Levels of PGC-1α, however, were 60 % lower in hypoxic muscle compared with that of chloride-supplemented normoxic animals and this was prevented by nitrate supplementation (Fig. 1g). Interestingly, PGC-1α levels were 50 % higher in the muscle of nitrate-supplemented normoxic rats compared with control animals (*P* ≤0.001). Thus, although the changes in PGC-1α were not associated with significant changes in citrate synthase activity, our findings nevertheless suggested that nitrate may exert effects on mitochondrial biogenesis via PGC-1α, and that this might become apparent with higher doses of nitrate.

Finally, to understand the energetic implications of these changes, we measured AMP, ADP and ATP levels in the soleus of these rats. Hypoxic exposure decreased ATP and increased AMP levels, indicating impaired energetics, whilst nitrate supplementation prevented this (Fig. 1h).

### Dietary nitrate enhances FA oxidation and mitochondrial biogenesis in the skeletal muscle of normoxic animals in a dose-dependent manner

Since nitrate supplementation was found to enhance FA oxidation in normoxic skeletal muscle, we focused on normoxic rats alone in order to establish whether the different effects observed on mitochondrial biogenesis and FA oxidation are dose dependent. Rats received the same SQC diet as before and water supplemented with 0 mM (control), 0.35 mM (low), 0.7 mM (medium), and 1.4 mM (high) sodium nitrate. As such, the medium dose group was equivalent to that received by the nitrate-supplemented normoxic group in the hypoxia study. Nitrate supplementation did not affect food or water intake, and body weight was unaffected (Table 1). Nitrate intake increased dose-dependently across the groups (Table 1), as did plasma nitrate levels; however, plasma nitrite levels only increased in the high dose group, as shown previously [30].

**Table 1:** The effects of 18 days supplementation with low (0.35 mM), medium (0.7 mM) and high (1.4 mM) doses of dietary nitrate on food, water and nitrate intakes, and body weight in rats, data = mean ± SEM, n = 5–6 per group

	Control	Low	Medium	High
Food intake (g/day)	30 ± 1	30 ± 1	30 ± 1	30 ± 1
Water intake (mL/day)	30 ± 3	35 ± 2	36 ± 3	32 ± 3
Calculated intake				
Nitrate (mg/kg/day)	1 ± 1	5 ± 2	8 ± 2	13 ± 1
Body weight				
Start (g)	265 ± 5	268 ± 6	270 ± 4	271 ± 3
End (g)	406 ± 8	420 ± 13	415 ± 9	404 ± 4

**Fig. 2:**
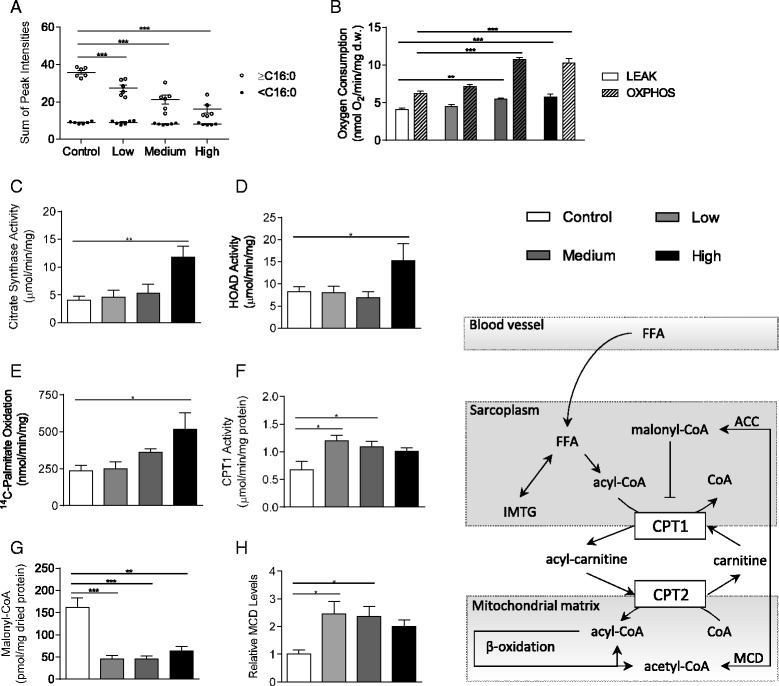
Dietary nitrate enhances fatty acid oxidation and mitochondrial biogenesis in skeletal muscle. (**a**) Fatty acid profile of soleus tissue divided into ≥ C16 (longer-chain) and < C16 (shorter-chain); (**b**) Respiration rates with palmitoyl-carnitine and malate substrates in permeabilized soleus muscle fibres; (**c**) Citrate synthase activity in soleus tissue; (**d**) ^14^C-Palmitate oxidation rates in mitochondrial isolates from soleus muscle; (**e**) 3-hydroxyacyl-CoA dehydrogenase activity in soleus tissue; (**f**) CPT1 activity in mitochondrial isolates from soleus muscle; (**g**) Malonyl-CoA levels in soleus tissue extracts; (**h**) Malonyl-CoA decarboxylase protein levels in soleus tissue. Data are represented as mean ± SEM, n = 4–6 per experimental group. * *P* ≤0.05; ** *P* ≤0.01; *** *P* ≤0.001

Next, we measured total FA concentrations in whole skeletal muscle, and found that shorter-chain FAs were the same in all groups, whilst longer-chain FAs (those ≥ C16) decreased dose-dependently with nitrate supplementation (Fig. 2a), suggesting that nitrate increased FA catabolism in a dose-dependent manner. Correspondingly, oxygen consumption of permeabilized soleus muscle fibres with 0.04 mM palmitoyl-carnitine and 5 mM malate also increased with nitrate supplementation (Fig. 2b), with LEAK state respiration increasing dose-dependently, yet OXPHOS respiration peaking at the medium dose (72 % greater than controls) and remaining elevated at the high dose.

To understand whether these effects might be attributed to changes in mitochondrial density or intra-mitochondrial changes, we measured citrate synthase and HOAD activities in whole skeletal muscle lysates from these rats. Citrate synthase (CS) activity was not altered by the low or medium doses of nitrate, but was 2.9-fold higher in rats receiving high-dose nitrate supplementation compared with controls (Fig. 2c). Meanwhile, HOAD activity showed a similar pattern, with no effect of low or medium doses of nitrate supplementation, but 86 % higher activity at the highest dose of nitrate (Fig. 2d). Thus, whilst the effects of high-dose nitrate supplementation might be attributed, at least in part, to mitochondrial biogenesis, it appears that at lower doses there is nevertheless an enhanced capacity for FA oxidation which is driven by intra-mitochondrial changes.

Therefore we further investigated the nitrate-enhanced FA oxidation in mitochondrial isolates using ^14^C-palmitate oxidation and CPT1 activity. ^14^C-palmitate oxidation per unit of mitochondrial protein was increased by nitrate supplementation, though this only reached statistical significance at the highest dose of nitrate (Fig. 2e). CPT1 activity, however, was increased by low and medium doses of nitrate, peaking at the lowest dose of nitrate used here and not reaching statistical significance at the highest dose (Fig. 2f). Tissue levels of malonyl-CoA, a negative regulator of CPT1, were decreased by 60–70 % at all doses of nitrate (Fig. 2g). This appeared to be due to an upregulation of MCD, since protein levels of MCD were elevated by nitrate supplementation in a similar fashion to CPT1 activity and were inversely related to tissue malonyl-CoA, peaking in the skeletal muscle of the low-dose-supplemented group and failing to reach significance at the higher dose (Fig. 2h).

### Dietary nitrate enhances FA oxidation in skeletal muscle via enhanced PPARα and PPARβ/δ transcriptional activity

Thus far, our data suggested that nitrate exerts dose-dependent effects on skeletal muscle FA oxidation, with intra-mitochondrial effects predominating at lower doses, including a release of inhibition on mitochondrial FA import via the CPT system, and a stimulation of mitochondrial biogenesis at higher doses. We next sought to investigate the downstream signalling/effector pathways mediating these nitrate-induced effects.

**Fig. 3:**
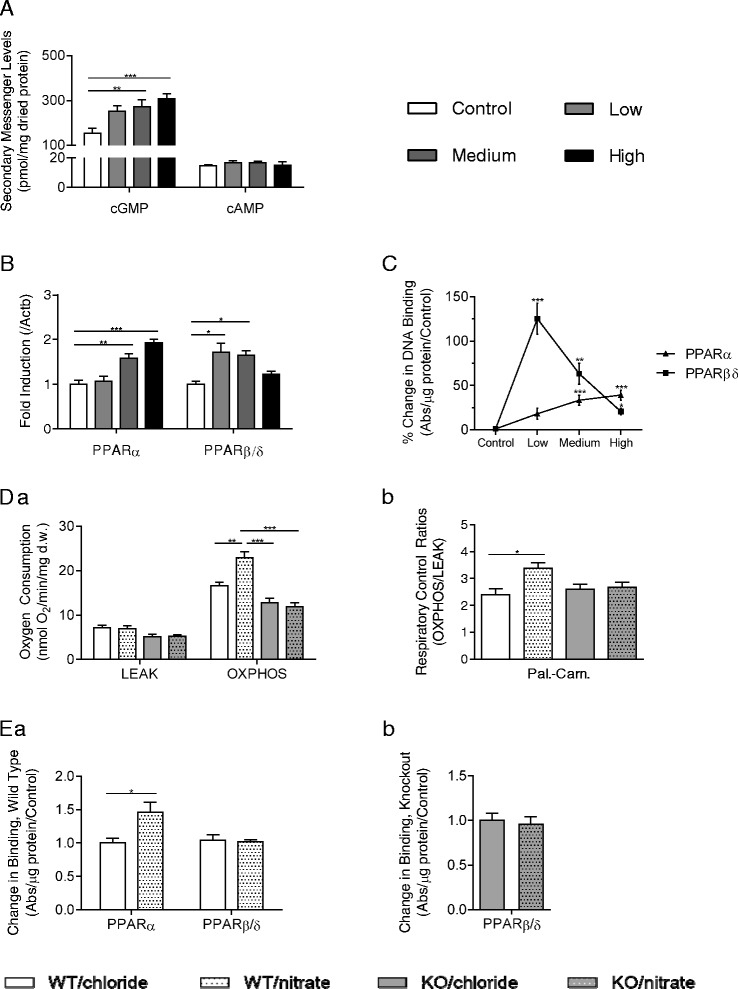
Dietary nitrate enhances fatty acid oxidation in skeletal muscle via enhanced PPARα and PPARβ/δ activity. (**a**) Levels of cAMP and cGMP in soleus extracts from rats supplemented with control (0 mM), low (0.35 mM), medium (0.7 mM) and high (1.4 mM) doses of dietary nitrate; (**b**) PPARα and PPARβ/δ expression in soleus tissue; (**c**) PPARα and PPARβ/δ DNA-binding in nuclear extracts of soleus; (**d**) Respiration rates and respiratory control ratio with palmitoyl-carnitine and malate substrates in permeabilized soleus muscle fibres from wild-type and PPARα^−/−^ mice; (**e**) PPARα and PPARβ/δ DNA-binding in nuclear extracts of soleus from wild-type mice and PPARα^−/−^ mice. Data are represented as mean ± SEM, n = 3–8 per experimental group. * *P* ≤0.05; ** *P* ≤0.01; *** *P* ≤0.001

NO, a putative product of nitrate reduction, is known to increase levels of the secondary messenger cGMP in tissue; therefore, we first measured cGMP and cAMP levels in the soleus of the rats. We found that nitrate elevated cGMP in a dose-dependent manner, whereas cAMP was unaffected by nitrate at any dose (Fig. 3a).

Since cGMP activates PPAR signalling [31] and many FA oxidation enzymes and regulators are transcriptional targets of PPARα and PPARβ/δ, we investigated the mechanism of these dose-dependent effects, focusing on PPAR-driven transcription. The expression of PPARα and PPARβ/δ in the soleus of rats supplemented with nitrate was analysed. We found that PPARα levels increased dose-dependently with nitrate supplementation, being 1.8-fold higher in the soleus of high-dose rats, compared with controls. Expression of PPARβ/δ was also increased with nitrate supplementation but peaked at the low dose, and was not significantly elevated in the soleus of high-dose rats (Fig. 3b). Correspondingly, PPARα DNA-binding activity increased dose-dependently with nitrate, whilst that of PPARβ/δ was enhanced by nitrate but peaked at the lowest dose (Fig. 3c). It therefore appears that the dose-dependence of the effects we previously observed relate to the divergent downstream effects of the different PPAR isoforms.

To further confirm that the effects of nitrate occur via PPARα activation, we switched our attention to PPARα^−/−^ mice, which, along with wild-type controls, were supplemented with 0.7 mM sodium nitrate (or sodium chloride) for 14 days. In agreement with studies in rats, we found that nitrate increased palmitoyl-carnitine respiration in the OXPHOS state by 38 % in wild-type mice. In PPARα^−/−^ mice, palmitoyl-carnitine respiration was lower than in wild-type mice, as expected, and was not affected by nitrate supplementation (Fig. 3d). Nitrate increased PPARα DNA-binding activity in wild-type mice, but PPARβ/δ DNA-binding activity did not increase in PPARα^−/−^ or wild-type mice with nitrate supplementation (Fig. 3e).

### Nitrate enhances FA oxidation capacity via NO-cGMP-PKG in C2C12 myotubes

Our data therefore suggested a role for enhanced PPARα transcriptional activity in the effects that nitrate exerts on FA oxidation. We hypothesized that this might occur via NO activation of sGC, leading to elevation of cGMP and hence downstream activation of PKG. We therefore sought to test this proposed mechanism in C2C12 myoblasts exposed to different concentrations of sodium nitrate.

**Fig. 4:**
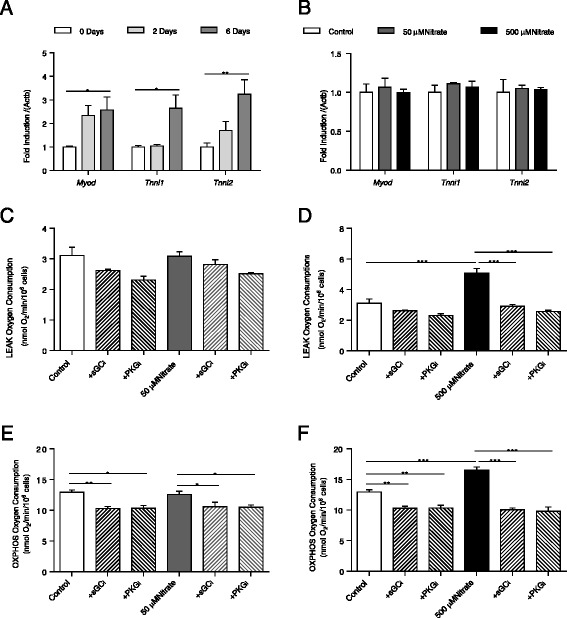
Nitrate enhances fatty acid oxidation capacity via NO-cGMP-PKG in C2C12 myotubes. (**a**) Muscle differentiation marker expression in C2C12 myoblasts cultured and differentiated over 6 days; (**b**) Muscle differentiation marker expression in C2C12 myocytes cultured and differentiated over 6 days in the presence of 0, 50 and 500 μM nitrate. See also Additional file 1: Figure S1. LEAK state respiration of palmitoyl-carnitine and malate substrates in permeabilized C2C12 myotubes cultured and differentiated over 6 days in the presence of (**c**) 0 or 50 μM nitrate or (**d**) 0 or 500 μM nitrate, in the presence or absence of sGC_i_ (ODQ, 1 μM) or PKG_i_ (KT5823, 1 μM). OXPHOS state respiration of palmitoyl-carnitine and malate substrates in permeabilized C2C12 myotubes cultured and differentiated over 6 days in the presence of (**e**) 0 or 50 μM nitrate or (**f**) 0 or 500 μM nitrate, in the presence or absence of sGC_i_ (ODQ, 1 μM) or PKG_i_ (KT5823, 1 μM). Data are represented as mean ± SEM, n = 4 repeats per condition. * *P* ≤0.05; ** *P* ≤0.01; *** *P* ≤0.001

Following induction of differentiation, cells expressed muscle differentiation markers (*MyoD*, *Tnni1* and *Tnni2*) after 6 days (Fig. 4a), and this was unaffected by any of the experimental conditions we later used; i.e. supplementation with nitrate alone at either 50 or 500 μM (Fig. 4b) or nitrate in the presence of inhibitors for sGC or PKG (Additional file 1: Figure S1). Nitrate supplementation at 50 μM did not alter LEAK state respiration in digitonin-permeabilized cells in the presence of palmitoyl-carnitine (Fig. 4c); however, this rate was enhanced by 500 μM nitrate – an effect prevented by both sGC and PKG inhibition (Fig. 4d). OXPHOS state respiration was also unaffected by 50 μM nitrate; however, both sGC and PKG inhibition decreased this respiration rate in C2C12 cells, whether supplemented with nitrate or not (Fig. 4e). At 500 μM, nitrate did enhance palmitoyl-carnitine respiration in the OXPHOS state (Fig. 4f) – an effect abrogated by sGC and PKG inhibition.

**Fig. 5:**
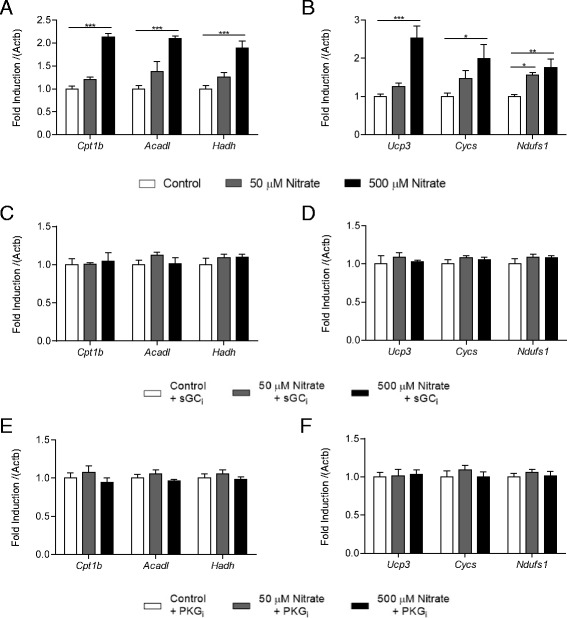
Nitrate enhances the expression of fatty acid oxidation enzymes and other mitochondrial proteins via NO-cGMP-PKG in C2C12 myotubes. (**a**) *Cpt1b*, *Acad1* and *Hadh*, and (**b**) *Ucp3*, *Cycs* and *Ndufs1* expression in C2C12 myotubes cultured and differentiated over 6 days in the presence of 0, 50 and 500 μM nitrate, (**c** and **d**) in the presence and absence of sGC_i_ (ODQ, 1 μM), and (**e** and **f**) in the presence and absence of PKG_i_ (KT5823, 1 μM). Data are represented as mean ± SEM, n = 4 repeats per condition. * *P* ≤0.05; ** *P* ≤0.01; *** *P* ≤0.001

We proceeded to investigate the effects of nitrate supplementation on the expression of FA oxidation enzymes and other mitochondrial proteins in C2C12 cells. We found that expression of *Cpt1b*, *Acadl* and *Hadh* (encoding CPT1B, long-chain acyl-CoA dehydrogenase and HOAD, respectively), was enhanced by 500 μM, but not 50 μM, nitrate supplementation (Fig. 5a), as was the expression of *Ucp3* and *Cycs* (encoding uncoupling protein 3 and cytochrome c, respectively), whilst expression of the complex I subunit *Ndufs1* was enhanced at both 50 and 500 μM nitrate (Fig. 5b). The effects of nitrate were time-dependent, reaching significance after 6 days of differentiation (Additional file 2: Figure S2). Notably, inhibition of sGC (Figs. 5c,d) and PKG (Figs. 5e,f) abolished all of these effects, confirming that the mechanism underlying the effects of nitrate on enhanced PPAR expression and transcriptional activity are mediated via the NO-cGMP-PKG signalling pathway.

**Fig. 6:**
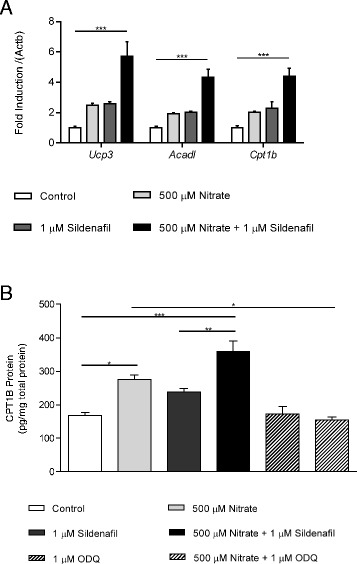
Nitrate and sildenafil cumulatively increase the expression of fatty acid oxidation enzymes and other mitochondrial proteins, and increase CPT1B protein levels. (**a**) *Ucp3*, *Acad1* and *Cpt1b* expression in C2C12 myotubes differentiated in the presence of 500 μM nitrate, 1 μM sildenafil, or both nitrate and sildenafil in combination. (**b**) CPT1B protein levels in C2C12 cells exposed to 500 μM nitrate, 1 μM sildenafil, nitrate and sildenafil in combination, 1 μM ODQ, and nitrate and ODQ in combination. Data are represented as mean ± SEM, n = 3 repeats per condition. * *P* ≤0.05; ** *P* ≤0.01; *** *P* ≤0.001

To further confirm the role of cGMP in mediating the effect of nitrate on FA oxidation, we treated C2C12 cells with 500 μM nitrate and/or 1 μM of the cGMP-specific phosphodiesterase 5 inhibitor, sildenafil, which prevents cGMP breakdown. As before, 500 μM nitrate increased expression of *Ucp3*, *Acadl* and *Cpt1b*, and a similar increase in expression of all three targets was seen with 1 μM sildenafil alone (Fig. 6a). As expected, 500 μM nitrate and 1 μM sildenafil in combination, acting to both increase cGMP production and decrease cGMP breakdown, produced a greater increase in expression of all three targets than either treatment alone (Fig. 6a).

Finally, we set out to confirm that the changes in *Cpt1b* expression were reflected at the protein level. Both 500 μM nitrate and 1 μM sildenafil increased CPT1B protein concentration in C2C12 cells, with a greater increase in CPT1B seen when nitrate and sildenafil were administered in combination (Fig. 6b). Meanwhile, treatment with the sGC inhibitor, 1H-[1,2,4]oxadiazolo[4,3-a]quinoxalin-1-one (ODQ), prevented the effect of nitrate on CPT1B concentration (Fig. 6b).

## Discussion

**Fig. 7:**
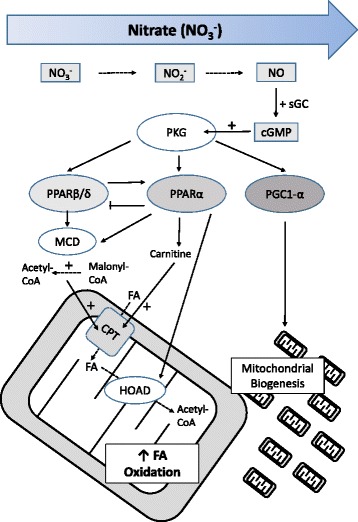
Schematic outlining the proposed mechanism underlying the effect of dietary nitrate on fatty acid (FA) oxidation and mitochondrial biogenesis in skeletal muscle. Nitrate (NO_3_^−^) is sequentially reduced to nitrite (NO_2_^−^) and nitric oxide (NO), which activates soluble guanylate cyclase. Thus, nitrate dose-dependently increases cellular cGMP, activating protein kinase G. At low doses of nitrate, PPARβ/δ levels increase, increasing expression of CPT1 and lowering malonyl-CoA, increasing FA oxidation. At intermediate doses of nitrate, induction of PPARα augments these changes and increases carnitine levels, further increasing FA oxidation. Meanwhile, PPARβ/δ expression is repressed by PPARα activation. At higher doses, increased expression of 3-hydroxyacyl CoA dehydrogenase occurs alongside elevated citrate synthase, indicating mitochondrial biogenesis, which further enhances muscle capacity for FA oxidation

Herein, we report that moderate doses of dietary inorganic nitrate increase the capacity for FA oxidation in skeletal muscle. The mechanism is dose-dependent, with increased expression and transcriptional activity of PPARβ/δ and PPARα occurring at lower nitrate concentrations, upregulating intra-mitochondrial pathways of FA oxidation, relieving inhibition of the CPT shuttle by malonyl-CoA via the upregulation of MCD, and increasing muscle carnitine concentrations. Notably, the same doses of nitrate did not increase FA oxidation in the muscle of PPARα^−/−^ mice. Increased mitochondrial volume density was observed at the highest dose of nitrate used in this study, supporting the capacity of muscle for complete FA oxidation. The underlying mechanism involves enhanced NO bioavailability, which activates sGC to increase muscle cGMP concentrations, subsequently activating PKG and leading to PPARα and (at higher doses) PGC-1α upregulation (Fig. 7). Indeed, in myocytes, inhibition of sGC or PKG prevented the nitrate-driven upregulation of PPARα-target genes and other mitochondrial proteins.

The excellent agreement in results across different techniques, experimental models and platforms provided a solid foundation for our study and allowed not only a comprehensive description of the effects of nitrate on muscle FA oxidation but also the elucidation of the underlying mechanism. For instance, the use of isolated rat muscle mitochondria allowed us to consider the changes in intra-mitochondrial FA oxidation pathways, whilst the use of permeabilized muscle fibres, protein levels and enzyme activities provided a picture of FA oxidation capacity at the tissue level. Meanwhile, targeted analysis of tissue metabolites (FA methyl esters to measure total FA content, carnitines/acyl-carnitines to indicate mitochondrial FA import, AMP/ADP/ATP to indicate energetic status, nitrogen oxides, and malonyl-CoA) supports the notion that changes in capacity measured ex vivo correspond to changes in FA oxidation in vivo. In order to establish underlying mechanisms, we again employed a number of approaches, including DNA-binding assays in nitrate-supplemented rats and wild-type and PPARα^−/−^ mice, plus cell respiration and gene expression analysis in cultured myocytes alongside the use of targeted inhibitors of upstream actors in our proposed pathway (sGC and PKG). The finding that nitrate enhanced FA oxidation in cultured myocytes indicates that effects are independent of changes in blood flow, and thus oxygen delivery, secondary to the vasodilatory effects of nitrogen oxides.

As with our previous studies of the effects of dietary nitrate on tissue metabolism and oxygen delivery [22, 23, 30], our use of a SQC diet and deionized water allowed us to acutely manipulate micronutrient concentrations in rats and mice, whilst accurately monitoring nitrate intake without restricting food or water intake. The nitrate interventions used were relatively short-term in duration (14 – 18 days) and although we would expect this to reflect the long-term effects of nitrate supplementation it would be worth confirming this. Whilst we have shown that nitrate still exerts these effects in hypoxic rats, it would be interesting to additionally investigate and assess the effectiveness of these mechanisms during other metabolic stresses (e.g. high-fat feeding, diabetes or exercise).

The effects of nitrate and hypoxia on FA oxidation and mitochondrial biogenesis appear to be tissue-specific. For instance, whilst we previously found that nitrate supplementation (at the medium dose used herein) modestly increased the capacity of rat cardiac mitochondria to oxidise palmitoyl-carnitine (by 25 %) and prevented the hypoxic suppression of FA oxidation [23], there was no such hypoxic suppression apparent in the soleus, and instead the dominant effect of nitrate was to double the capacity for palmitoyl-carnitine respiration. In the mammalian heart, FA oxidation is the major source of ATP generation under normal conditions [32], whereas skeletal muscle utilises carbohydrate and FA more evenly [33], so there is more scope to increase FA oxidation in skeletal muscle. It is perhaps surprising that there was no significant loss of mitochondria and/or FA oxidation capacity in the hypoxic rat soleus, given that such effects are well-documented in human skeletal muscle following sustained exposure to hypoxia [34–36]; however, it should be noted, this does not occur in humans with more acute exposure [35, 37]. The relative preservation of FA oxidation in hypoxia may also be a particular feature of the soleus, a highly-oxidative, type 1 muscle, which contrasts with the mixed fibre-type *vastus lateralis* commonly biopsied for use in human studies. It is notable, however, that whilst there are variations in mitochondrial function between different muscles of the mouse hindlimb, it is mouse soleus that most closely resembled human quadriceps in terms of the coupling control of electron transport during FA oxidation [38].

Meanwhile, in WAT (and primary adipocytes), nitrate and hypoxia act synergistically in promoting the browning response, with the greatest expression of PGC-1α, CPT1 and, particularly, UCP1 occurring under hypoxic/nitrate-supplemented conditions [22]. It is possible that hypoxia systemically augments the production of NO from dietary nitrate by sequential reduction, with the expression and nitrate reductase activity of xanthine oxidoreductase increasing under hypoxic conditions [39]. The increased availability of NO in low oxygen conditions may, however, be offset in skeletal muscle and heart, by the HIF-dependent downregulation of PPARα and PGC-1α [34], preventing downstream metabolic effects. Indeed, in the absence of hypoxia, there are striking parallels between the effects of nitrate on rat WAT and skeletal muscle, particularly regarding the dose-dependency of the changes observed. For instance, citrate synthase activity increased in both tissues in a dose-dependent manner, whilst CPT1 protein/activity peaked at the lowest dose of nitrate used (0.35 mM) as did UCP1 in WAT [22].

The dose dependent effects of nitrate we report herein may be attributable to differential effects on the expression of PPAR isoforms and PGC-1α. At the lowest dose of nitrate used, there was no induction of PPARα, but the greatest expression of PPARβ/δ and enhanced PPARβ/δ-DNA binding, alongside elevated CPT1 activity. Whilst this dose was not associated with a significant increase in mitochondrial palmitoyl-carnitine respiration or muscle ^14^C-palmitate oxidation ex vivo, we saw decreased long-chain FAs in the muscles of these rats, suggesting more subtle effects on in vivo FA oxidation at this dose. At the medium dose of nitrate, PPARα expression was induced in addition to PPARβ/δ and this resulted in increased palmitoyl-carnitine respiration and ^14^C-palmitate oxidation, alongside a larger decrease in total long-chain FAs. Whilst there was no increase in citrate synthase activity at this dose, PGC-1α levels were increased. At the highest dose of nitrate used, PPARβ/δ expression and PPARβ/δ-DNA binding fell below that of lower doses, though PPARα expression and DNA-binding activity was highest. Our data suggest a suppression of PPARβ/δ by PPARα activation, an effect previously described in rat brain astrocytes [40]. Alongside an increase in mitochondrial density at the highest dose, the elevated PPARα levels supported the greatest rate of ^14^C-palmitate oxidation and resulted in the lowest levels of total long-chain FAs, although, interestingly, mitochondrial palmitoyl-carnitine respiration (representing β-oxidation) was no higher than at the medium dose. These findings show similarities with high-fat fed mice that have undertaken exercise-training to elevate PGC-1α expression [14]. There was surprisingly good agreement between the effects of nitrate at these doses between rats and mice in the present study. NO production rates [41] and circulating nitrate/nitrite concentrations [42] are similar between rats and humans, but lower than those in mice [43]. Our data, however, demonstrate that nitrate supplementation at the intermediate dose results in increased PPARα DNA-binding (though not PPARβ/δ DNA-binding) in mice too.

The intermediate dose of nitrate used herein matches that shown to have metabolic effects in studies of human muscle [44] and which can be achieved in humans via a slight modification of the diet, e.g. via a modest increase in consumption of leafy green vegetables such as spinach and beetroot [45]. In humans, this dose was found to decrease the oxygen cost of exercise [46], improve mitochondrial efficiency via a lowering of LEAK state respiration [44], and to lower whole-body resting metabolic rate [45]. These effects were associated with decreased muscle expression of the putative uncoupler adenine nucleotide translocase, but also a strong trend towards decreased UCP3 expression [44]. Our findings in rodents do seem to contradict some of these effects in humans. However, beyond the obvious difference in species, some other key differences between the studies should be noted, along with some similarities. Firstly, our rats were maintained on a standardised version of normal rat chow, which is low in fat content, whereas the humans essentially consumed a regular diet, albeit avoiding foodstuffs high in nitrate [44, 45]. Therefore, rats would have conceivably had a greater scope to increase their capacity for FA oxidation compared to humans as PPAR activation via the diet would have been lower at baseline, whilst the rats were also sedentary and would therefore be expected to have relatively low muscle mitochondrial contents at baseline. In addition, the mitochondrial respiration studies of human *vastus lateralis* employed pyruvate and malate as substrates [44], as opposed to the FA derivative palmitoyl-carnitine that we used here, and as such they were not optimised to see differences in FA oxidation. Interestingly, however, although LEAK state respiration increased in the rats with nitrate supplementation in our study, respiratory control ratios (OXPHOS/LEAK) did in fact increase in both rats and wild-type mice, indicating improved mitochondrial coupling and thus enhanced efficiency with a higher rate of oxidative phosphorylation. Finally, we have not measured whole-body oxygen consumption in these rats, and it would be interesting to do so, but previous work from our group with the livers of rats receiving the same doses of nitrate showed a dose-dependent fall in HIF-1 activation, suggesting a better matching of oxygen supply and demand despite decreased circulating haemoglobin, and thus possibly indicating decreased oxygen consumption or improved mitochondrial efficiency, at least in this tissue [30].

Our finding of enhanced skeletal muscle FA oxidation capacity with moderate consumption of dietary nitrate now deserves further investigation in healthy human subjects, as well as those with metabolic disease, with further investigation in animal models of metabolic syndrome. A similar dose of nitrate was previously found to have anti-obesity and insulin-sensitizing effects in the eNOS^−/−^ mouse, which exhibits a metabolic syndrome-like phenotype [21], and it would be interesting to see whether nitrate exerts similarly-protective effects in obese and insulin-resistant rodents where the primary defect is not one of NO-production, e.g. diet-induced obesity or leptin-deficiency/resistance. In combination with our previous finding that nitrate stimulates the browning of WAT, however, our work suggests that nitrate supplementation holds promise as a potential therapy for metabolic disease.

## Conclusions

We have found that a moderate dose of dietary nitrate enhances skeletal muscle FA oxidation capacity by promoting intra-mitochondrial pathways of FA oxidation and, at higher doses, mitochondrial biogenesis. The underlying mechanism is dose-dependent, and occurs via enhanced NO bioavailability and increased muscle cGMP levels, leading to the upregulation of PPARα and PGC-1α.

## Methods

All procedures involving live animals were carried out by a licence holder in accordance with UK Home Office regulations, and underwent review by the University of Cambridge Animal Welfare and Ethical Review Committee. Some, but not all, of the rats used in this work were also used in separate, but parallel studies of oxygen consumption by other tissues [22, 23] and oxygen delivery [30], with a view to reducing the total numbers of animals used in accordance with UK Home Office best practice. Where relevant data (e.g. nitrate intakes and plasma [NO_x_]) has been reported previously, we refer to the previous paper and thus have not duplicated data.

### Animal studies

Male Wistar rats (273 ± 2 g; n = 40) acquired from Charles River (UK) were maintained on a standardized quality-controlled chow to normalize micronutrient levels (RM1(E)SQC, Special Diets Services, UK; 55 % carbohydrate, 3 % fat, 15 % protein). After 12 days acclimatization, animals received either 0.7 mM NaNO_3_ (nitrate group) or NaCl (control group; both ultra-pure, Sigma-Aldrich) in distilled water (n = 20/group). After 4 days, half of each group were transferred to hypoxia chambers (13 % O_2_; 20 air changes/hr) (PFI Systems Ltd, UK; n = 10/group). All animals remained in these conditions, with ad libitum access to food and NaNO_3_/NaCl-supplemented water for 14 days. Animals were housed pairwise in conventional cages at controlled humidity and temperature with a 12-h light/dark cycle.

To establish a dose–response study, male Wistar rats (269 ± 2 g; n = 24*)* were housed under normoxia conditions otherwise identical to those above, on standardized rodent chow, with access to distilled water ad libitum. After 12 days acclimatization, rats received distilled water or water supplemented with NaNO_3_ (0.35, 0.7, 1.4 mM; n = 6/group) ad libitum for 18 days. Wild-type 129Ev/Sv and PPARα^−/−^ mice (2–3 months of age), a gift of Dr Frank Gonzalez (National Cancer Institute, Bethesda MD, USA), were housed under identical conditions, and received distilled water or water supplemented with 0.7 mM NaNO_3_ (n = 8/group) ad libitum for 14 days.

### C2C12 studies

C2C12 cells (Sigma-Aldrich) were cultured in 1 mL Dulbecco’s Modified Eagle’s Medium (DMEM; 4.5 g/L glucose, L-glutamine, NaHCO_3_ and pyridoxine-HCl) supplemented with 10 % fetal bovine serum and 1× penicillin/streptomycin (100 U/mL and 100 μg/mL, respectively) in 12-well plates (Millipore, USA) at 37 °C in 5 % CO_2_. Medium was changed daily, and upon reaching confluence, cells were maintained in differentiation medium for 6 days (DMEM supplemented with 2 % horse serum and 1× penicillin/streptomycin). Differentiation medium was changed daily for the first 3 days, and every 12 h for the remaining 3 days. After 2 days of differentiation, cells were also supplemented with 0, 50 or 500 μM nitrate. In inhibitor experiments, cells were additionally incubated with either 1 μM ODQ (sGC inhibitor) for the final 2 days of differentiation, 1 μM KT5823 (PKG inhibitor; Santa Cruz Biotechnology Inc., USA) for the final 3 days, 1 μM sildenafil (cGMP-specific phosphodiesterase 5 inhibitor) for the final 4 days, or 1 μL dimethyl sulfoxide as control. In the time-course study of nitrate effects on selected gene expression, cells were supplemented with 500 μM nitrate at 2, 4 and 6 days before collection, whilst ‘0 days’ received no nitrate and acted as a control.

To produce sufficient cells for CPT1B protein quantification, C2C12 cells were instead grown and differentiated in collagen I-coated T75 flasks (Millipore, USA), using the same medium-change procedure and nitrate/inhibitor concentrations as above. Cells were lysed with 1 mL radio-immunoprecipitation buffer (Sigma-Aldrich) supplemented with protease inhibitor (Complete mini protease inhibitor cocktail, Roche, Germany) and the protein concentration quantified using the Pierce BCA Protein Assay kit (Thermo Scientific).

For respirometry analysis, the medium was removed and 0.5 mL trypsin (10× trypsin-EDTA, Invitrogen) added to separate cells from the well, 0.5 mL differentiation medium was added to each well to inactivate trypsin, and the resulting solution pipetted into a microcentrifuge tube. From the microcentrifuge tubes, 100 μL of solution were added to 900 μL PBS and the cell number counted. Tubes containing the remaining cells were centrifuged at 600 × *g* for 5 min to pellet the cells; the medium was removed and the pellet gently resuspended in 600 μL respiration medium.

### Nitrate and nitrite levels

Nitrate was quantified using a dedicated HPLC system (ENO-20, Eicom; Tokyo, Japan), employing sequential ion chromatography, online reduction to nitrite using a cadmium column, and post-column-derivatization with a modified Griess reagent, as described in previously published protocols [47].

### Metabolic profiling

Aqueous and organic metabolites were extracted from soleus muscle as described previously [23].

Aqueous extracts were reconstituted in 100 μL 70:30 acetonitrile:water before metabolites were measured using hydrophilic interaction liquid chromatography-mass spectrometry. Multiple reaction monitoring in positive ion mode was used with the following optimised mass transitions: malonyl-CoA, 854.0 > 347.1; cGMP, 346.1 > 152.1; cAMP, 330.1 > 136.1; AMP, 348.03 > 135.90; ADP, 427 > 135.90; ATP, 507.91 > 135.90. Malonyl-CoA, cGMP, cAMP, AMP, ADP and ATP had separately optimised declustering potentials and collision energies as follows: 41 V, 23 eV; 81 V, 41 eV; 71 V, 31 eV; 95.0 V, 18 eV; 30.0 V, 30.0 eV; 30.0 V, 30.0 eV; and 35.0 V, 15.0 eV, respectively. Other optimisation parameters common to each species were ion spray voltage 5.5 kV and temperature 500 °C. A 5-μL injection volume was analysed on a 5500 QTRAP mass spectrometer (AB Sciex, Toronto, Canada) attached to an Acquity ultra-performance liquid chromatography system (UPLC; Waters Corporation, MA, USA). Chromatographic separation was achieved using a 100 mm × 2.1 mm × 1.7 μm BEH amide hydrophilic interaction liquid chromatography column (Waters) with 30 % aqueous containing 10 mM ammonium acetate (pH 9 with ammonium hydroxide) increasing to 50 % aqueous over 5 min at a flow rate of 600 μL/min. The column was re-equilibrated for a further 3 min at cessation of the gradient. Malonyl-CoA was normalised to a universally ^13^C- and ^15^N-labelled glutamate internal standard (Cambridge Isotope Laboratories Inc., USA) and compared with a 6-point malonyl-CoA calibration line over the 10 nM to 50 μM concentration range due to non-linearity at low nanomolar concentrations. cGMP data were normalised to the same internal standard and compared with an 8-point cGMP calibration line with concentrations ranging from 1 nM to 50 μM. cAMP data were normalised to the same internal standard and compared with an 8-point cAMP calibration line with concentrations ranging from 1 nM to 50 μM.

Organic extracts were reconstituted in 400 μL 1:1 chloroform:methanol and mixed thoroughly. When fully dissolved, 100 μL of each sample were transferred to a 3.5 mL glass vial; the remaining 300 μL were dried overnight in a fume hood and frozen at −20 °C and 650 μL of 1:1 chloroform:methanol, D25-tridecanoic acid as internal standard to a final concentration of 21 μM, and 125 μL 10 % BF_3_/methanol were added to each sample. This derivatisation procedure converts FAs to fatty acid methyl esters. The vials were then vortexed, and incubated for 90 min at 80 °C. After cooling, 500 μL HPLC-grade water and 1 mL hexane were added, and the vials thoroughly mixed by vortexing. The upper, organic, layer was transferred to a 2 mL GC vial and allowed to dry overnight in a fume hood.

FAME samples were reconstituted in 200 μL hexane and a 2 μL injection run on a Trace GC Ultra coupled to a Trace DSQ II single quadrupolar mass spectrometer (Thermo Scientific). The oven parameters were as follows: initial temperature 60 °C; ramp 15 °C/min to 150 °C; and ramp 2 4 °C/min to 230 °C (total run time of 28 min). The transfer line from the oven to the mass spectrometer was heated to 240 °C and the inlet to 230 °C. The mass spectrometer was run in split mode, with a flow of 10 mL/min and a split ratio of 8. Chromatographic separation was achieved using a 30 m × 0.25 mm × 0.25 μm 70 % cyanopropyl polysilphenlyene-siloxane TR-FAME column (Thermo Scientific) with 1.2 mL/min helium at constant flow as carrier. Positive ion mode data were collected after an initial 4 min delay with three scans per second in the 50–650 m/*z* range. Chromatograms were processed using the Xcalibur software suite (version 2.2; Thermo Scientific). Individual peaks were integrated and subsequently normalised to total peak intensity. Peaks were assigned based on fragmentation patterns and matched to the National Institute of Standards and Technology (NIST, USA) library. Peak identification was supported by comparing retention times and fragmentation patterns to the Supelco 37-Component FAME Mix (Sigma-Aldrich) and to the American Oil Chemists’ Society archive of methyl ester derivatives (AOCS, USA).

### Respirometry

Respirometry was performed on 2–5 mg saponin- permeabilized soleus muscle fibre bundles at 37 °C using Clark-type oxygen electrodes (Strathkelvin Instruments, Strathkelvin, UK), essentially as described [23]. Briefly, 0.04 mM palmitoyl-carnitine and 5 mM malate were supplied as substrate (LEAK state) before oxygen consumption was stimulated by the addition of 2 mM ADP (OXPHOS state). Muscle fibres were recovered from the electrode chamber to allow normalisation of oxygen consumption rates to dry muscle mass. For C2C12 studies, cells suspended in 500 μL of respiration medium were added to the Clark-type electrode chambers for analysis, along with 0.02 % w/v digitonin to permeabilize cells, and respiration carried out as it was for muscle fibres, with rates normalised to number of cells.

### CPT1 activity and palmitate oxidation assays

Mitochondria were isolated from soleus muscle from rats according to published protocols [48]. CPT1 activity was determined in soleus muscle mitochondrial isolates using ^3^H-carnitine according to previously published protocols [49]. The assay buffer contained 117 mM Tris–HCl, 0.28 mM reduced glutathione, 4.4 mM MgCl_2_, 16.7 mM KCl, 2.2 mM KCN, 300 μM palmitoyl-CoA, 5 mM carnitine, 4.4 mM ATP, 40 mg/L rotenone, 0.5 % BSA, and 1 μCi ^3^H-carnitine. The reaction was initiated with the addition of 20 μL of mitochondrial homogenates and incubated for 8 min at 37 °C. The reaction was then terminated by the addition of 60 μL of HCl. The palmitoyl-^3^H-carnitine formed during the reaction was separated according to previously published protocols [50, 51] and the radioactivity counted to determine CPT1 activity. The reaction is linear in rats for 8 min at protein concentrations of <0.5 mg mitochondrial protein [46] and all assays were carried out within these limits.

Palmitate oxidation rates were measured in soleus muscle mitochondrial isolates using ^14^C-palmitate according to previously published methods [52, 53]. Briefly, modified Krebs-Ringer buffer (115 mM NaCl, 2.6 mM KCl, 1.2 mM KH_2_PO_4_, 10 mM NaHCO_3_, 10 mM HEPES, pH 7.4) supplemented with 5 mM ATP, 1 mM NAD^+^, 0.5 mM carnitine, 0.1 mM coenzyme A, 25 μM cytochrome *c*, and 0.5 mM malate were incubated at 37 °C in a 20 mL glass scintillation vial. A suspended microcentrifuge tube containing 150 μL of benzethonium hydroxide were placed inside the vial to trap the ^14^CO_2_ produced during the reaction. Mitochondria were added to the system, which was then sealed with a rubber cap. The reaction was initiated by the addition of 6:1 palmitate:BSA complex (containing 1 μCi of ^14^C-palmitate) to a final palmitate concentration of 100 μM via a syringe through the rubber cap. The vial was incubated for 30 min at 37 °C before termination with 50 μL of HClO_4_. The microcentrifuge tube containing the benzethonium hydroxide and trapped ^14^CO_2_ was then removed, transferred to a scintillation vial, and the radioactivity counted.

### Enzyme analysis

Frozen tissues were powdered under liquid nitrogen with a mortar and pestle and homogenized in potassium phosphate buffer (100 mM KH_2_PO_4_, 5 mM EDTA, 0.1 % Triton X-100, pH 7.2) using a polytron homogenizer. HOAD activity was assayed according to a published protocol [54]. The assay buffer contained 50 mM imidazole (pH 7.4), 0.1 mM acetoacetyl-CoA, 0.15 mM NADH, and 0.1 % Triton X-100. NADH absorbance was monitored at 340 nm for 3 min.

CS was assayed according to a previously published protocol [55]. The assay buffer contained 20 mM Tris (pH 8.0), 0.1 mM 5,5’-dithiobis-(2-nitrobenzoic acid) and 0.3 mM acetyl-CoA. The reaction was initiated by the addition of 0.5 mM oxaloacetate (omitted from controls), and absorbance monitored at 412 nm for 3 min.

### PPAR binding assays

Frozen tissue was crushed using a liquid nitrogen-cooled pestle and mortar, and homogenates prepared from which the nuclear subcellular fraction was isolated using a commercial kit (Cayman Chemical Company, MI, USA). Ice-cold hypotonic buffer was added to crushed tissue in a 5 μL:1 mg ratio. The solution was homogenised using a polytron before being incubated on ice for 15 min. After incubation, samples were centrifuged at 1,000 × *g* for 10 min at 4 °C to pellet cell debris, and the supernatant containing the cytosolic fraction transferred to a pre-chilled micro-centrifuge tube. The pellet was resuspended in 500 μL hypotonic buffer and incubated on ice. After 15 min, 50 μL 10 % Nonidet P-40 were added and the samples centrifuged at 14,000 × *g* for 30 s at 4 °C. The supernatant was combined with the cytosolic fraction from the previous step and frozen at −80 °C. The pellet was resuspended in 50 μL ice-cold extraction buffer, vortexed vigorously for 15 s and subsequently rocked on a shaking platform for 15 min on ice. The vortexing and shaking were repeated, and the samples centrifuged at 14,000 × *g* for 10 min at 4 °C. The supernatant was transferred to pre-chilled tubes, flash frozen and stored at −80 °C until use. A small aliquot was used to quantify protein concentration using a spectrophotometer.

PPARα and β/δ transcriptional activity were assayed in nuclear extracts using a commercial transcription factor kit (Cayman Chemical Company, MI, USA). The wells of a plate were coated by a double-stranded DNA (dsDNA) sequence containing the peroxisome proliferator response element. By utilising nuclear extracts, only binding of proteins within the nucleus are quantified, which effectively represents activated PPARs. Nuclear extracts and all kit reagents were allowed to equilibrate to room temperature before use. To the blank and non-specific binding wells 100 μL of transcription factor assay buffer were added. In the competitor dsDNA wells, 80 μL transcription factor assay buffer were added followed by 10 μL PPAR competitor dsDNA. In the positive control wells, 90 μL of transcription factor assay buffer were added followed by 90 μL of positive control. Finally, 90 μL of transcription factor assay buffer were added to each sample well followed by 10 μL nuclear extract. The plate was covered and incubated overnight at 4 °C without agitation. The wells were then emptied and washed five times with 200 μL of wash buffer, with care taken on the final wash to remove residual buffer. To each well, except the blanks, 100 μL of either PPARα or PPARβ/δ primary antibody solution were added and the plate covered and incubated for 1 h at room temperature without agitation. The wash step was repeated as above and any residual wash buffer carefully removed. To each well, except the blanks, 100 μL of horseradish peroxidase (HRP)-conjugated secondary antibody were added and the plate covered and incubated for 1 h at room temperature without agitation. The wells were washed with 200 μL of wash buffer as above, before 100 μL of developing solution was added and the plate incubated for 30 min at room temperature with gentle shaking. After incubation, 100 μL stop solution were added to each well and the absorbance read at 450 nm within 5 min.

### Gene expression analysis

Total RNA was purified from crushed, frozen muscle using an RNeasy Mini Kit (QIAgen) according to the manufacturer’s specifications. RNA concentration was quantified at 260 nm using a SmartSpecPlus spectrophotometer (Bio-Rad). For analysis of steady-state mRNA levels, the relative abundance of transcripts of interest was assessed by quantitative-PCR in SYBR Green FastStart Universal Master Mix (Applied Biosystems) with a StepOnePlus detection system (Applied Biosystems). QuantiTect primer assays for rat *Ppara* and *Pparbd* were obtained from QIAgen. Expression levels were normalized to Actb using the ∆C_T_ method, and subsequently to ‘normoxia/chloride’ group in the hypoxia study and to the ‘control’ group in the dose–response study, to give fold-changes.

For gene analysis in C2C12 cells, RNA extraction was performed as above, with the single difference being that 450 μL of lysis buffer were added directly to the wells, and subsequently pipetted onto the spin columns for purification. Production of cDNA and RT-qPCR analysis of *Myod*, *Tnni1*, *Tnni2*, *CptIb*, *Acadl*, *Hadh*, *Ucp3*, *Cycs*, and *Ndufs1* expression proceeded as above, using QuantiTect primer assays obtained from QIAgen.

### Immunoblotting

Immunoblotting for citrate synthase, PGC-1α and malonyl-CoA decarboxylase was performed on soleus muscle lysates according to published protocols [56]. Membranes were incubated in primary antibody solution containing 1:1000 rabbit polyclonal IgG raised against CS, PGC-1α or MCD (all Abcam, UK) for 2 h at room temperature (CS and PGC-1α) or overnight at 4 °C (MCD). After washing with TBS-T for 2 h with a solution change every 15 min, membranes were incubated in secondary antibody solution containing 1:1750 goat anti-rabbit IgG, conjugated to HRP for 1 h, before visualisation using ECL-plus and quantification as previously described [56].

### CPT1B enzyme-linked immunosorbent assay

CPT1B protein concentration was measured using a commercial mouse CPT1B ELISA (MyBioSource, USA), according to the manufacturer’s specifications, in cell lysates prepared as detailed above. Samples (diluted 20 times) and standards were incubated in duplicate on a 96-well plate containing an immobilised antibody to mouse CPT1B for 2 h at 37 °C. After unbound substances were washed away, biotin conjugated to a secondary antibody raised against CPT1B was added, and the plate incubated for 1 h at 37 °C. After further washes, a streptavidin-HRP conjugate was added and incubated for 1 h at 37 °C, followed by further washes to remove unbound conjugate. A substrate solution was then added to the wells and incubated, protected from light, for 30 min at 37 °C. Diluted HCl was added to stop the enzymatic reaction, and finally the optical density was measured at 450 nm with readings at 540 nm subtracted to account for optical imperfections in the plate. Data were collated and normalised to total protein in the original sample.

### Statistics

Results are expressed as mean ± SEM. Analysis of variance (ANOVA) was used to determine significant differences across the four groups of the hypoxia and dose–response studies. Data were collated in Excel before 1- or 2-way analysis of variance (ANOVA) was used to determine significant differences across experimental groups (Graphpad, Instat). Bonferroni post-hoc analysis was used for multiple analysis of selected groups, where appropriate. Differences were considered significant when *P* <0.05.

### Availability of supporting data

All data supporting the results of this article are available in an online Additional file 3.

## Additional files

Additional file 1: Figure S1. Muscle differentiation marker expression in C2C12 myoblasts cultured and differentiated over 6 days in the presence of 0, 50 and 500 μM nitrate, and (**A**) in the presence and absence of sGC_i_ (1H-[1,2,4] oxadiazolo[4,3-a]quinoxalin-1-one (ODQ), 1 μM) and (**B**) in the presence and absence of PGK_i_ (KT5823, 1 μM). Data are represented as mean ± SEM, n = 4 repeats per condition. (PDF 52 kb) Additional file 2: Figure S2.
*Ucp3*, *Acadl* and *Cpt1b* expression in C2C12 myoblasts cultured and differentiated over 6 days in the presence of 500 μM nitrate. Data are represented as mean ± SEM, n = 3 repeats per condition. *** *P* ≤0.001. (PDF 24 kb) Additional file 3:
**Supporting data.** (XLSX 45.3 kb)

## Abbreviations

cAMP: cycline adenosine monophosphate
cGMP: cyclic guanosine monophosphate
CPT: carnitine palmitoyl-transferase
CS: citrate synthase
eNOS: endothelial nitric oxide synthase
FA: fatty acid
HIF: hypoxia-inducible factor
HOAD: 3-hydroxyacyl CoA dehydrogenase
HRP: horseradish peroxidase
MCD: malonyl-CoA decarboxylase
NO: nitric oxide
OXPHOS: oxidative phosphorylation
PGC-1α: PPARγ coactivator 1α
PKG: cGMP activated protein kinase
PPAR: peroxisome proliferator-activated receptor
sGC: soluble guanylyl cyclase
SQC: standardised quality controlled
UCP: uncoupling protein
WAT: white adipose tissue

## References

[1] Kelley DE (2005). Skeletal muscle fat oxidation: timing and flexibility are everything. J Clin Invest.

[2] Ukropcova B, McNeil M, Sereda O, de Jonge L, Xie H, Bray GA (2005). Dynamic changes in fat oxidation in human primary myocytes mirror metabolic characteristics of the donor. J Clin Invest.

[3] Mootha VK, Lindgren CM, Eriksson KF, Subramanian A, Sihag S, Lehar J (2003). PGC-1alpha-responsive genes involved in oxidative phosphorylation are coordinately downregulated in human diabetes. Nat Genet.

[4] Simoneau JA, Veerkamp JH, Turcotte LP, Kelley DE (1999). Markers of capacity to utilize fatty acids in human skeletal muscle: relation to insulin resistance and obesity and effects of weight loss. FASEB J.

[5] Shulman GI (2014). Ectopic fat in insulin resistance, dyslipidemia, and cardiometabolic disease. N Engl J Med.

[6] Kiens B, Alsted TJ, Jeppesen J (2011). Factors regulating fat oxidation in human skeletal muscle. Obes Rev.

[7] Barish GD, Narkar VA, Evans RM (2006). PPAR delta: a dagger in the heart of the metabolic syndrome. J Clin Invest.

[8] Thoresen GH, Hessvik NP, Bakke SS, Aas V, Rustan AC (2011). Metabolic switching of human skeletal muscle cells in vitro. Prostaglandins Leukot Essent Fat Acids.

[9] Lee CH, Olson P, Hevener A, Mehl I, Chong LW, Olefsky JM (2006). PPARdelta regulates glucose metabolism and insulin sensitivity. Proc Natl Acad Sci U S A.

[10] Roberts LD, Murray AJ, Menassa D, Ashmore T, Nicholls AW, Griffin JL (2011). The contrasting roles of PPARdelta and PPARgamma in regulating the metabolic switch between oxidation and storage of fats in white adipose tissue. Genome Biol.

[11] Koves TR, Ussher JR, Noland RC, Slentz D, Mosedale M, Ilkayeva O (2008). Mitochondrial overload and incomplete fatty acid oxidation contribute to skeletal muscle insulin resistance. Cell Metab.

[12] Muoio DM, Newgard CB (2006). Obesity-related derangements in metabolic regulation. Annu Rev Biochem..

[13] Puigserver P, Spiegelman BM (2003). Peroxisome proliferator-activated receptor-gamma coactivator 1 alpha (PGC-1 alpha): transcriptional coactivator and metabolic regulator. Endocr Rev.

[14] Koves TR, Li P, An J, Akimoto T, Slentz D, Ilkayeva O (2005). Peroxisome proliferator-activated receptor-gamma co-activator 1alpha-mediated metabolic remodeling of skeletal myocytes mimics exercise training and reverses lipid-induced mitochondrial inefficiency. J Biol Chem.

[15] Nisoli E, Clementi E, Paolucci C, Cozzi V, Tonello C, Sciorati C (2003). Mitochondrial biogenesis in mammals: the role of endogenous nitric oxide. Science.

[16] Miyashita K, Itoh H, Tsujimoto H, Tamura N, Fukunaga Y, Sone M (2009). Natriuretic peptides/cGMP/cGMP-dependent protein kinase cascades promote muscle mitochondrial biogenesis and prevent obesity. Diabetes.

[17] Mitschke MM, Hoffmann LS, Gnad T, Scholz D, Kruithoff K, Mayer P (2013). Increased cGMP promotes healthy expansion and browning of white adipose tissue. FASEB J.

[18] Hoffmann LS, Etzrodt J, Willkomm L, Sanyal A, Scheja L, Fischer AW (2015). Stimulation of soluble guanylyl cyclase protects against obesity by recruiting brown adipose tissue. Nat Commun..

[19] Kapil V, Milsom AB, Okorie M, Maleki-Toyserkani S, Akram F, Rehman F (2010). Inorganic nitrate supplementation lowers blood pressure in humans: role for nitrite-derived NO. Hypertension.

[20] Bryan NS, Fernandez BO, Bauer SM, Garcia-Saura MF, Milsom AB, Rassaf T (2005). Nitrite is a signaling molecule and regulator of gene expression in mammalian tissues. Nat Chem Biol.

[21] Carlstrom M, Larsen FJ, Nystrom T, Hezel M, Borniquel S, Weitzberg E (2010). Dietary inorganic nitrate reverses features of metabolic syndrome in endothelial nitric oxide synthase-deficient mice. Proc Natl Acad Sci U S A.

[22] Roberts LD, Ashmore T, Kotwica AO, Murfitt SA, Fernandez BO, Feelisch M (2015). Inorganic nitrate promotes the browning of white adipose tissue through the nitrate-nitrite-nitric oxide pathway. Diabetes.

[23] Ashmore T, Fernandez BO, Branco-Price C, West JA, Cowburn AS, Heather LC (2014). Dietary nitrate increases arginine availability and protects mitochondrial complex I and energetics in the hypoxic rat heart. J Physiol.

[24] Horscroft JA, Murray AJ (2014). Skeletal muscle energy metabolism in environmental hypoxia: climbing towards consensus. Extreme Physiol Med.

[25] Heather LC, Cole MA, Tan JJ, Ambrose LJ, Pope S, Abd-Jamil AH (2012). Metabolic adaptation to chronic hypoxia in cardiac mitochondria. Basic Res Cardiol.

[26] Narravula S, Colgan SP (2001). Hypoxia-inducible factor 1-mediated inhibition of peroxisome proliferator-activated receptor alpha expression during hypoxia. J Immunol.

[27] Stephens FB, Wall BT, Marimuthu K, Shannon CE, Constantin-Teodosiu D, Macdonald IA (2013). Skeletal muscle carnitine loading increases energy expenditure, modulates fuel metabolism gene networks and prevents body fat accumulation in humans. J Physiol.

[28] Whitmer JT, Idell-Wenger JA, Rovetto MJ, Neely JR (1978). Control of fatty acid metabolism in ischemic and hypoxic hearts. J Biol Chem.

[29] Oram JF, Wenger JI, Neely JR (1975). Regulation of long chain fatty acid activation in heart muscle. J Biol Chem.

[30] Ashmore T, Fernandez BO, Evans CE, Huang Y, Branco-Price C, Griffin JL (2015). Suppression of erythropoiesis by dietary nitrate. FASEB J.

[31] Wang J, Yang K, Xu L, Zhang Y, Lai N, Jiang H (2013). Sildenafil inhibits hypoxia-induced transient receptor potential canonical protein expression in pulmonary arterial smooth muscle via cGMP-PKG-PPARgamma axis. Am J Respir Cell Mol Biol.

[32] Lopaschuk GD, Ussher JR, Folmes CD, Jaswal JS, Stanley WC (2010). Myocardial fatty acid metabolism in health and disease. Physiol Rev.

[33] Frayn KN (2010). Metabolic regulation: a human perspective.

[34] Murray AJ (2009). Metabolic adaptation of skeletal muscle to high altitude hypoxia: how new technologies could resolve the controversies. Genome Med.

[35] Levett DZ, Radford EJ, Menassa DA, Graber EF, Morash AJ, Hoppeler H (2012). Acclimatization of skeletal muscle mitochondria to high-altitude hypoxia during an ascent of Everest. FASEB J.

[36] Jacobs RA, Siebenmann C, Hug M, Toigo M, Meinild AK, Lundby C (2012). Twenty-eight days at 3454-m altitude diminishes respiratory capacity but enhances efficiency in human skeletal muscle mitochondria. FASEB J.

[37] Jacobs RA, Boushel R, Wright-Paradis C, Calbet JA, Robach P, Gnaiger E (2013). Mitochondrial function in human skeletal muscle following high-altitude exposure. Exp Physiol.

[38] Jacobs RA, Diaz V, Meinild AK, Gassmann M, Lundby C (2013). The C57Bl/6 mouse serves as a suitable model of human skeletal muscle mitochondrial function. Exp Physiol.

[39] Jansson EA, Huang L, Malkey R, Govoni M, Nihlen C, Olsson A (2008). A mammalian functional nitrate reductase that regulates nitrite and nitric oxide homeostasis. Nat Chem Biol.

[40] Aleshin S, Grabeklis S, Hanck T, Sergeeva M, Reiser G (2009). Peroxisome proliferator-activated receptor (PPAR)-gamma positively controls and PPARalpha negatively controls cyclooxygenase-2 expression in rat brain astrocytes through a convergence on PPARbeta/delta via mutual control of PPAR expression levels. Mol Pharmacol.

[41] Siervo M, Stephan BC, Feelisch M, Bluck LJ (2011). Measurement of in vivo nitric oxide synthesis in humans using stable isotopic methods: a systematic review. Free Radic Biol Med.

[42] Pannala AS, Mani AR, Spencer JP, Skinner V, Bruckdorfer KR, Moore KP (2003). The effect of dietary nitrate on salivary, plasma, and urinary nitrate metabolism in humans. Free Radic Biol Med.

[43] Milsom AB, Fernandez BO, Garcia-Saura MF, Rodriguez J, Feelisch M (2012). Contributions of nitric oxide synthases, dietary nitrite/nitrate, and other sources to the formation of NO signaling products. Antioxid Redox Signal.

[44] Larsen FJ, Schiffer TA, Borniquel S, Sahlin K, Ekblom B, Lundberg JO (2011). Dietary inorganic nitrate improves mitochondrial efficiency in humans. Cell Metab.

[45] Larsen FJ, Schiffer TA, Ekblom B, Mattsson MP, Checa A, Wheelock CE (2014). Dietary nitrate reduces resting metabolic rate: a randomized, crossover study in humans. Am J Clin Nutr.

[46] Larsen FJ, Weitzberg E, Lundberg JO, Ekblom B (2007). Effects of dietary nitrate on oxygen cost during exercise. Acta Physiol.

[47] Bryan NS, Rassaf T, Maloney RE, Rodriguez CM, Saijo F, Rodriguez JR (2004). Cellular targets and mechanisms of nitros(yl)ation: an insight into their nature and kinetics in vivo. Proc Natl Acad Sci U S A.

[48] Campbell SE, Tandon NN, Woldegiorgis G, Luiken JJ, Glatz JF, Bonen A (2004). A novel function for fatty acid translocase (FAT)/CD36: involvement in long chain fatty acid transfer into the mitochondria. J Biol Chem.

[49] McGarry JD, Brown NF (2000). Reconstitution of purified, active and malonyl-CoA-sensitive rat liver carnitine palmitoyltransferase I: relationship between membrane environment and malonyl-CoA sensitivity. Biochem J.

[50] Le Belle JE, Harris NG, Williams SR, Bhakoo KK (2002). A comparison of cell and tissue extraction techniques using high-resolution 1H-NMR spectroscopy. NMR Biomed.

[51] Starritt EC, Howlett RA, Heigenhauser GJ, Spriet LL (2000). Sensitivity of CPT I to malonyl-CoA in trained and untrained human skeletal muscle. Am J Physiol Endocrinol Metab.

[52] Bezaire V, Heigenhauser GJ, Spriet LL (2004). Regulation of CPT I activity in intermyofibrillar and subsarcolemmal mitochondria from human and rat skeletal muscle. Am J Physiol Endocrinol Metab.

[53] Morash AJ, McClelland GB (2011). Regulation of carnitine palmitoyltransferase (CPT) I during fasting in rainbow trout (Oncorhynchus mykiss) promotes increased mitochondrial fatty acid oxidation. Physiol Biochem Zool.

[54] McClelland GB, Dalziel AC, Fragoso NM, Moyes CD (2005). Muscle remodeling in relation to blood supply: implications for seasonal changes in mitochondrial enzymes. J Exp Biol.

[55] Houle-Leroy P, Garland T, Swallow JG, Guderley H (2000). Effects of voluntary activity and genetic selection on muscle metabolic capacities in house mice Mus domesticus. J Appl Physiol (1985).

[56] Heather LC, Cole MA, Lygate CA, Evans RD, Stuckey DJ, Murray AJ (2006). Fatty acid transporter levels and palmitate oxidation rate correlate with ejection fraction in the infarcted rat heart. Cardiovasc Res.

