# Supplementary figures and images for: Nitrate enhances skeletal muscle fatty acid oxidation via a nitric oxide-cGMP-PPAR-mediated mechanism
Source: BMC Biol. 2015 Dec 22;13:110. doi: 10.1186/s12915-015-0221-6 (PMC4688964; doi:10.1186/s12915-015-0221-6)

A

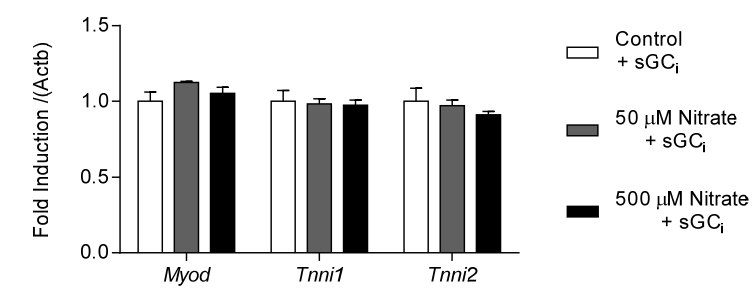

B

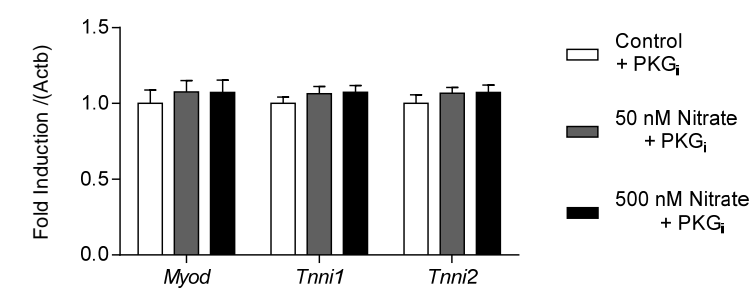

Supplement: Additional file 1: Figure S1. — Muscle differentiation marker expression in C2C12 myoblasts cultured and differentiated over 6 days in the presence of 0, 50 and 500 μM nitrate, and (A) in the presence and absence of sGCi (1H-[1,2,4] oxadiazolo[4,3-a]quinoxalin-1-one (ODQ), 1 μM) and (B) in the presence and absence of PGKi (KT5823, 1 μM). Data are represented as mean ± SEM, n = 4 repeats per condition. (PDF 52 kb) [file 12915_2015_221_MOESM1_ESM.pdf]

A

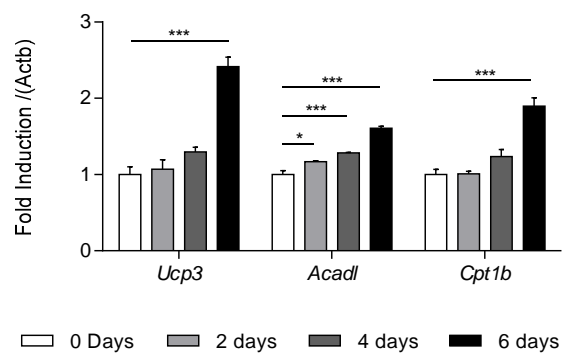

Supplement: Additional file 2: Figure S2. — Ucp3, Acadl and Cpt1b expression in C2C12 myoblasts cultured and differentiated over 6 days in the presence of 500 μM nitrate. Data are represented as mean ± SEM, n = 3 repeats per condition. *** P ≤0.001. (PDF 24 kb) [file 12915_2015_221_MOESM2_ESM.pdf]
